# Effects of smoking cessation on biological monitoring markers in urine

**DOI:** 10.1186/s41021-020-00165-z

**Published:** 2020-09-11

**Authors:** Yuya Kawasaki, Yun-Shan Li, Yuko Ootsuyama, Kazuhiko Nagata, Hiroshi Yamato, Kazuaki Kawai

**Affiliations:** 1 Department of Environmental Oncology, Institute of Industrial Ecological Sciences, University of Occupational and Environmental Health, Japan, 1-1 Iseigaoka, Yahatanishi-ku, Kitakyushu, Fukuoka, 807-8555 Japan; 2 Nagata Medical Clinic, 4-3-1 Takasu Higashi, Wakamatsu-ku, Kitakyushu, Fukuoka, 808-0144 Japan; 3 Department of Health Development, Institute of Industrial Ecological Sciences, University of Occupational and Environmental Health, Japan, 1-1 Iseigaoka, Yahatanishi-ku, Kitakyushu, Fukuoka, 807-8555 Japan; 4 Center for Stress-related Disease Control and Prevention, University of Occupational and Environmental Health, Japan, 1-1 Iseigaoka, Yahatanishi-ku, Kitakyushu, Fukuoka, 807-8555 Japan

**Keywords:** Nicotine, Cotinine, 4-(methylnitrosamino)-1-(3-pyridyl)-1-butanol (NNAL), 7-methylguanine (m^7^Gua), 8-hydroxy-2′-deoxyguanosine (8-OHdG), Smoking cessation

## Abstract

**Introduction:**

Urinary nicotine and cotinine levels are often measured as biomarkers for tobacco smoke exposure. However, these biomarkers are not appropriate to evaluate the effects of quitting smoking for several days, because of their short half-lives. In this study, we focused on the changes in the urinary 4-(methylnitrosamino)-1-(3-pyridyl)-1-butanol (NNAL) levels of 55 patients in a smoking cessation program, because of the long half-life. At the same time, urinary 7-methylguanine (m^7^Gua) and 8-hydroxy-2′-deoxyguanosine (8-OHdG), as DNA damage markers of cigarette smoking, were also measured.

**Results:**

In the subjects who completed the quit-smoking program (18 subjects out of 55), the urinary nicotine and cotinine levels decreased to 1.7 and 0.2% at 8 weeks after the first visit to the clinic. By contrast, the NNAL levels decreased to 12.3% at 8 weeks after quitting smoking. During the same period, the urinary m^7^Gua levels significantly decreased, from 27.32 μg/mg creatinine to 14.17 μg/mg creatinine by the elimination of subjects who showed increased levels of NNAL during the smoking cessation program. The 8-OHdG levels were also reduced within the same period, but were not significantly different. From the all data analysis, the urinary levels of cotinine and NNAL positively correlated with the level of m^7^Gua.

**Conclusions:**

NNAL may be an appropriate exposure marker for evaluating the smoking status of patients in a smoking cessation program. The urinary cotinine and NNAL levels positively correlated with the m^7^Gua levels.

## Introduction

Tobacco smoke contains more than 5000 chemicals and over 70 types of carcinogens [1, 2]. Smoking has been established as a risk factor for many common cancers [3]. The percentage of smokers is decreasing each year in Japan (from 24.2% in 2006 to 17.8% in 2018); however, it still remains high, especially among men (29.0% in men vs. 8.1% in women) [4]. Smoking cessation is one of the most effective interventions to prevent cancer. At smoking cessation programs in hospitals and clinics, the abstinence status is usually biochemically confirmed by an expiratory carbon monoxide (CO) concentration below 9 ppm. However, due to the short half-life of CO (2 h), many people who smoked over 24 h before the test could be misclassified as quitters [5]. In addition to CO, biochemical verification of tobacco abstinence can be obtained by measuring the cotinine levels in urine, plasma and saliva. Cotinine is the main metabolite of nicotine and has a longer half-life (18 h) than CO and nicotine (2 h) [6]. In measurements of tobacco use, cotinine has relatively high sensitivity and specificity, as compared to CO levels. Therefore, it is a reliable indicator of recent nicotine intake [7]. However, for patients undergoing nicotine replacement therapy (NRT) using nicotine gum and nicotine patches, cotinine cannot be used as an indicator of smoking cessation because nicotine and cotinine are present at the same levels as smokers [8–12]. On the other hand, 4-(methylnitrosamino)-1-(3-pyridyl)-1-butanol (NNAL) in urine has been reported as an exposure indicator that is not affected by NRT [13, 14]. NNAL is a tobacco-specific nitrosamine and a metabolite of the carcinogenic 4-(methylnitrosamino)-1-(3-pyridyl)-1-butanone (NNK) in humans [3]. Many studies have investigated the urinary NNAL levels in smokers and secondhand smokers [15, 16]. As urine contains quantitatively significant NNK metabolites, the NNAL levels in urine are critically useful in studies of human exposure to tobacco smoke. Urinary NNAL has a much longer half-life (10–40 days) than urinary cotinine and can be detected even a few weeks after smoking [6]. Therefore, NNAL is a possible candidate for an indicator of quitting smoking. However, only a few studies have used urinary NNAL as an indicator of smoking cessation in clinical settings.

Tobacco smoke contains many carcinogens that can lead to DNA methylation and oxidation. A metabolite of NNK and NNAL, methanediazonium ion, reacts with DNA to form methyl DNA base adducts, including 7-methylguanine (m^7^Gua) and O^6^-methyl deoxyguanine [17]. m^7^Gua is removed from DNA by a glycosylase to produce an apurinic site [18]. As the result, m^7^Gua is excreted into the urine. The apurinic site is a frequent cause of mutations in mammalian cells [19, 20]. Current smokers had higher m^7^Gua levels in their lung DNA [21]. A correlation between urinary m^7^Gua levels and urinary NNAL levels has been reported [22].

Oxidative damage of DNA by reactive oxygen species leads to the production of 8-hydroxy-2′-deoxyguanosine (8-OHdG), a specific biomarker of oxidative stress [23–25]. Several studies reported that urinary 8-OHdG levels [26–28] and salivary 8-hydroxyguanine levels [29, 30] correlated with smoking. Cao et al. [31] reported that the levels of 8-OHdG in bronchoalveolar lavage fluid were associated with the Tumor Node Metastasis stage, indicating that oxidative DNA damage is a marker for the development of lung cancer.

The purpose of this study is to investigate the reductions of cigarette smoking exposure markers (nicotine, cotinine and NNAL) and DNA damage markers (m^7^Gua and 8-OHdG) in patients participating in a smoking cessation program.

## Materials and methods

### Subjects and urine sample collection

A total of 55 subjects (36 male and 19 female, ages 25–68) from a smoking cessation clinic in Japan participated in the study. After excluding samples because of only a single visit or the use of only heated tobacco products, a total of 42 subjects (26 male and 16 female) were selected for the analysis (Table 1).

**Table 1:** Participants in this study

	Mean ± SD		
	Male	Female	Total
Ages (years)	44.6 ± 12.7	42.8 ± 10.3	43.9 ± 11.8
n	26	16	42

Subjects visited the clinic 2 to 5 times during the 12-week treatment period, and urine samples were collected at the first visit and at weeks 2 and 8 after quitting smoking. The number of subjects who participated up to the second visit (2 weeks) was 42 (Group 1; 26 male and 16 female). The number of subjects who participated up to the third visit (8 weeks) was 18 (Group 2; 11 male and 7 female). At each clinic visit, 10 mL urine samples were collected in polypropylene centrifuge tubes and stored at − 20 °C until analysis. The study protocol was approved by the Ethics Committee of Medicine and Medical Care, University of Occupational and Environmental Health, Japan.

### Chemicals

(−)-Nicotine (≥98%), (−)-cotinine (≥98%), and Type H-1 β-glucuronidase (208,400 units/g solid) were obtained from Sigma Aldrich Inc. (St. Louis, MO). Isolute SLE+ column cartridges were purchased from Biotage (Uppsala, Sweden). Chloroform (≥99.7%) and acetic acid (≥99.7%) were purchased from FUJIFILM Wako Pure Chemicals Co., Inc. (Osaka, Japan). Acetonitrile (≥99.8%), 1 mol/L ammonium acetate solution (LC/MS grade) and distilled water (LC/MS grade) were purchased from Kanto Chemical Co., Inc. (Tokyo, Japan). NNAL (100 μg/mL in acetonitrile), DL-methyl-D_3_-cotinine (100 μg/mL in acetonitrile), and 1, 2′, 3′, 4′, 5′, 6′-^13^C_6_-NNAL (100 μg/mL in acetonitrile) were obtained from Cambridge Isotope Laboratories, Inc. (Tewksbury, MA). The stock solutions (each 100 μg/mL) of nicotine, cotinine, and NNAL were prepared in acetonitrile. DL-methyl-D_3_-cotinine (10 μg/mL) and ^13^C_6_-NNAL (20 ng/mL) stock solutions were also prepared in acetonitrile. The working solutions were prepared using serial dilutions of the stock solutions with a 10% (v/v) acetonitrile solution containing 10 mM ammonium acetate.

### Sample preparation

Frozen urine samples were thawed at room temperature. Urine (500 μL) was mixed with 500 μL of acetate buffer (50 mM, pH 4.0), followed by the addition of 2 μL of DL- methyl-D_3_-cotinine and ^13^C_6_-NNAL stock solutions as internal standards. β-Glucuronidase (1000 U/500 μL of urine) was then added and the solution was incubated at 37 °C for 15 h. The mixture was loaded onto Isolute SLE+ column cartridges (Biotage) and allowed to adsorb on the diatomaceous earth supported material for 10 min, followed by elution with 6 mL of chloroform. The extract was evaporated to dryness at 40 °C under a continuous flow of nitrogen. The residue was dissolved in 200 μL of a 10% (v/v) acetonitrile solution containing 10 mM ammonium acetate. The solution was filtered through a pretreatment filter. The filtered solution was divided into 100 μL aliquots. One was diluted 5–20-fold with a 10% acetonitrile solution containing 10 mM ammonium acetate, for the measurement of urinary nicotine and cotinine. The other was used for urinary NNAL measurement without dilution.

### Liquid chromatography and mass spectrometry conditions

Analyses of urinary nicotine, cotinine and NNAL were conducted using an HPLC (UltiMate 3000, Thermo Fisher Scientific, Yokohama, Japan) coupled to a hybrid quadrupole-Orbitrap mass spectrometer (Thermo Scientific Q Exactive Focus) with heated electrospray ionization (HESI-II). The sample separation was achieved on an Acclaim™ 120 C18 (2.1 mm × 50 mm, 3 μm, Thermo Scientific, Sunnyvale, CA) column with a flow rate of 0.3 mL/min and a column temperature of 30 °C. Mobile phase A was 10 mM ammonium formate and mobile phase B was acetonitrile. The following linear gradient program was used for the separation, with a total run time of 15 min. The percentage of B solvent (acetonitrile) changed as follows: 0 min, 5%; 8 min, 32%; 8.1–11 min, 95%; 11.1–15 min, 5%. For measurements, the injection volumes were 2 μL for nicotine and cotinine, and 5 μL for NNAL. The ESI source was set to a heater temperature of 300 °C and the sheath gas and auxiliary gas pressures were set to 50 and 15 arbitrary units, respectively. The ion spray voltage was set to 2.5 kV, with a capillary temperature of 250 °C, and the S-lens RF level was 40. Data were acquired in the parallel reaction monitoring (PRM) mode. In this mode, a single precursor ion [M + H]^+^ was selected in the quadrupole with an isolation width of 2.0 m/z. After fragmentation in the higher energy collision-induced dissociation (HCD) cell, the resulting MS/MS product ions were detected in the Orbitrap detector at a resolution of 70,000. The most abundant ion was used for quantification.

### Calibration curves

The calibration standards were prepared at concentrations of 10, 50, 100, 250, and 500 ng/mL nicotine, and 5, 10, 50, 100, 250, and 500 ng/mL cotinine. Each standard solution was then spiked with 100 ng/mL of DL-methyl-D_3_-cotinine. The NNAL calibration curve was generated based on concentrations of 10, 50, 100, 250, and 500 pg/mL and each standard solution was spiked with 200 pg/mL of ^13^C_6_-NNAL. The calculations of limit of detection (LOD) and limit of quantification (LOQ) were applied to 3σ and 10σ criteria. If the value (before correction by the dilution ratio and urinary creatinine level) calculated from each calibration curve was less than the LOQ, then it was set to 0 level.

### Analyses of urinary 8-OHdG and m^7^Gua

Urinary m^7^Gua and 8-OHdG concentrations were determined by the previously described method [32]. Briefly, a human urine sample was mixed with the same volume of a dilution solution containing the ribonucleoside marker, 8-hydroxyguanosine (8-OHG). A 20 μL aliquot of the diluted urine sample was injected into HPLC-1 (MCI GEL CA08F, 7 μm, 1.5 × 120 mm; elution, 2% acetonitrile in 0.3 mM sulfuric acid, 50 μL/min, 65 °C), via the guard column (1.5 × 40 mm), and the chromatograms were recorded by a Gilson UV detector (UV/VIS-155 with 0.2 mm light path cell). Creatinine and m^7^Gua were detected at 235 and 305 nm, respectively. The 8-OHdG fraction was collected, depending on the relative elution position from the peak of the added marker, 8-OHG, and was automatically injected into the HPLC-2 column. The 8-OHdG fraction was fractionated by the HPLC-2 column (GL Science Inc., Inertsil ODS-3, 3 μm, 4.6 × 250 mm; elution, 10 mM sodium phosphate buffer [pH 6.7] containing 5% methanol and an antiseptic reagent MB [100 μL/L], 1 mL/ min, 45 °C). The 8-OHdG was detected by a Coulochem II EC detector (ESA Inc., Chemsford, MA, USA) with a guard cell (5020) and an analytical cell (5011) (applied voltages: guard cell, 350 mV; E1, 100 mV; E2, 300 mV).

### Statistical methods

The values of each biomarker were compared with the median, because the data did not follow a normal distribution. Statistical analyses were performed using GraphPad Prism, version 7.04 (GraphPad Software, San Diego, CA, USA). Data were tested for normality using Shapiro-Wilk’s test. Non-parametric tests were used because all variables were not normally distributed. Two-sided *p* values less than 0.05 were considered significant.

## Results

### Detection sensitivity of urinary tobacco exposure markers

**Fig. 1:**
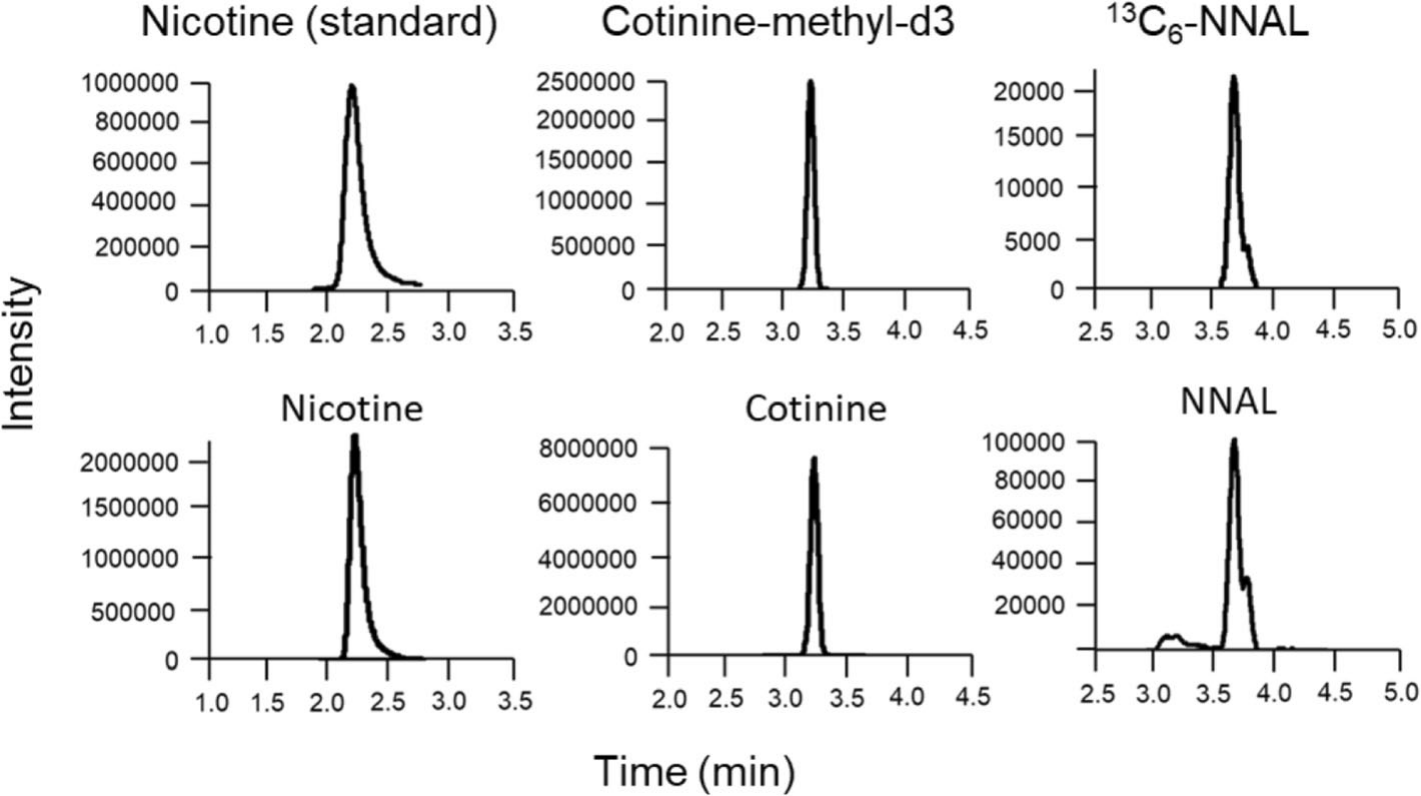
Chromatograms of smoking exposure markers in urine. Upper row: authentic samples, Lower row: urine sample

The lower detection limits for nicotine, cotinine, and NNAL were determined to be 0.79 ng/mL, 0.21 ng/mL, and 1.85 pg/mL, respectively. The lower limits of quantification for nicotine, cotinine, and NNAL were 2.62 ng/mL, 0.71 ng/mL, and 6.17 pg/mL, respectively. The correlation coefficients (r^2^) were > 0.99 in all cases. Representative chromatograms of these exposure markers in urine from a smoker are shown in Fig. 1.

### Urinary biomarker levels after smoking cessation

**Fig. 2:**
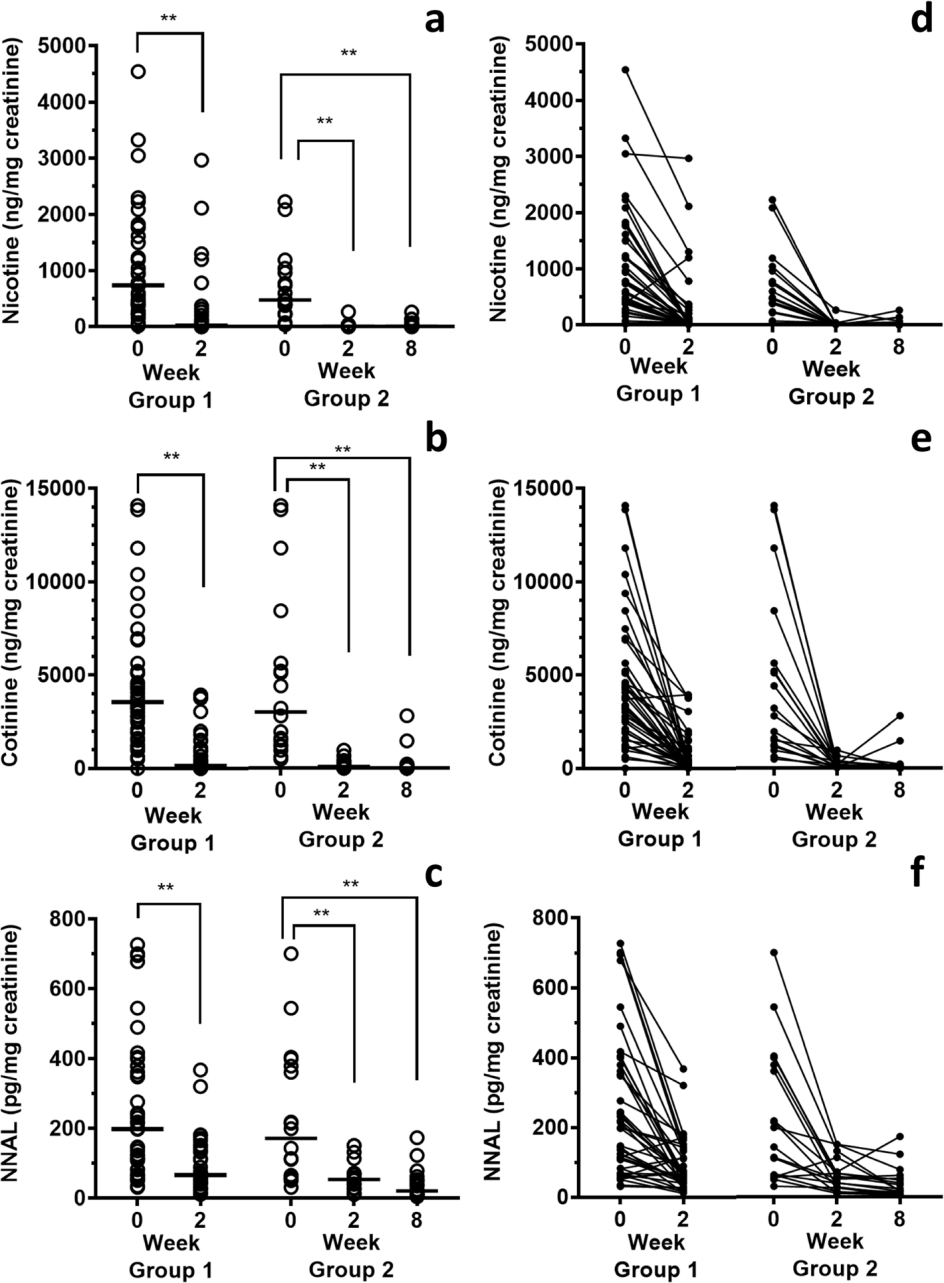
Changes in urinary tobacco exposure biomarker levels by smoking cessation. Horizontal bars indicate medians. Group 1: The subjects who participated in the quit-smoking program for 2 weeks (*n* = 42, Wilcoxon signed-rank test). Group 2: The subjects who completed the quit-smoking program (*n* = 18, Friedman’s test followed by Dunn’s multiple comparisons test). ** *p* < 0.01. Individual changes in biomarkers are indicated by the lines in (**d** – **f**)

**Fig. 3:**
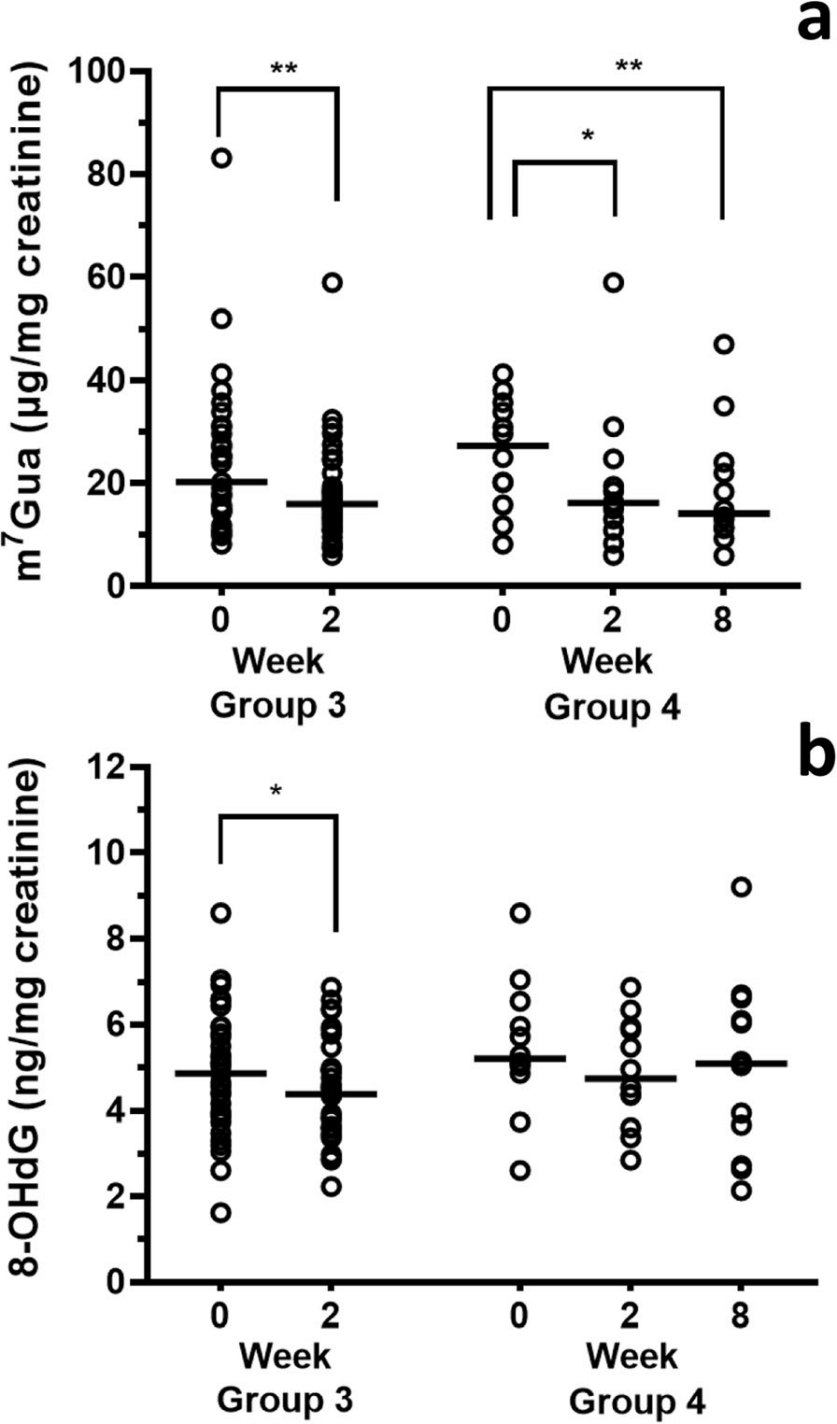
Changes in urinary m^7^Gua (**a**) and 8-OHdG (**b**) levels by smoking cessation in the subjects with continuous decreases in NNAL levels throughout the smoking cessation period. Horizontal bars indicate median values. Group 3: The subjects participated in the quit-smoking program up to 2 weeks (*n* = 37, Wilcoxon signed-rank test). Group 4: The subjects participated in the quit-smoking program up to 8 weeks (*n* = 12, Friedman’s test followed by Dunn’s multiple comparisons test). * *p* < 0.05; ** *p* < 0.01

**Fig. 4:**
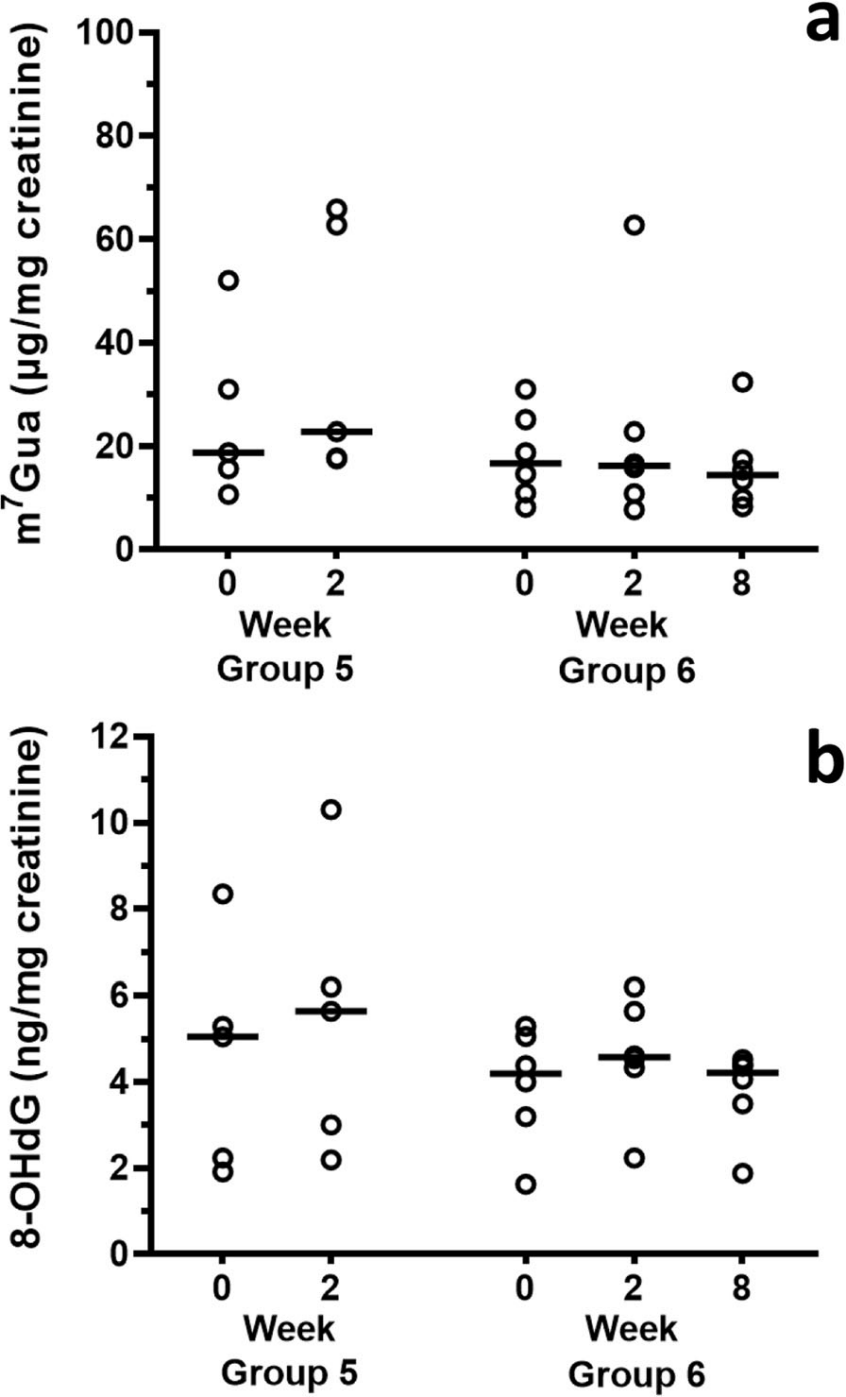
Changes in urinary m^7^Gua (**a**) and 8-OHdG (**b**) levels by smoking cessation in the subjects with increased NNAL levels at some point in the smoking cessation program. Horizontal bars indicate median values. Group 5: The subjects with increased NNAL levels at 2 weeks (*n* = 5). Group 6: The subjects who participated in the quit-smoking program for up to 8 weeks and had increased NNAL levels at some point (*n* = 6, Friedman’s test followed by Dunn’s multiple comparisons test). There are no significant differences from baseline

Urinary levels of cigarette smoking markers decreased clearly with time after smoking cessation for 2 and 8 weeks (Fig. 2). Among the subjects who completed the quit-smoking program (Group 2 in Fig. 2), the median values of the urinary nicotine levels (minimum-maximum) at the initial visit to the tobacco cessation clinic, and at 2 and 8 weeks later were 477.2 (36.3–2226.5) ng/mg creatinine, 8.7 (not detected (n.d.) – 264.9) ng/mg creatinine, and 8.2 (1.9–263.1) ng/mg creatinine. The urinary cotinine levels at the same times were 3006.0 (496.4–14,084.5) ng/mg creatinine, 102.7 (2.3–969.8) ng/mg creatinine, and 6.1 (n.d. - 2803.7) ng/mg creatinine. The urinary NNAL levels were 171.7 (31.0–700.9) pg/mg creatinine, 53.9 (10.9–151.4) pg/mg creatinine, and 21.1 (4.4–174.0) pg/mg creatinine. The nicotine, cotinine, and NNAL levels are represented as the total of the free and glucuronidated forms. The individual changes of each biomarker are shown in Fig. 2d-f. Looking at each individual, the urinary NNAL levels of 9 subjects (5 subjects in Group 1 and 6 subjects in Group 2, two subjects overlapped) increased at some points during smoking cessation, as compared to the previous medical examination. Although the rates of the NNAL increases were not markedly high, in order to evaluate the effects of quitting smoking on the DNA damage markers, those subjects were eliminated from the following group analysis and monitored separately. Consequently, the DNA damage markers in urine, as the early adverse health effect markers, were analyzed separately for those 9 individuals. In the analysis excluding those subjects, the DNA damage markers in urine decreased with time after smoking cessation (Fig. 3). In the subjects who completed the smoking cessation program (Group 4 in Fig. 3), the median values (with minimum-maximum) of the urinary m^7^Gua levels at the initial visit to the tobacco cessation clinic, and at 2 and 8 weeks later were 27.32 (8.21–41.30) μg/mg creatinine, 16.17 (6.05–58.94) μg/mg creatinine, and 14.17 (6.02–47.06) μg/mg creatinine. The urinary 8-OHdG levels at the same times were 5.21 (2.61–8.60) ng/mg creatinine, 4.75 (2.85–6.87) ng/mg creatinine, and 5.09 (2.14–9.20) ng/mg creatinine, respectively. In the case of all subjects (Group 3 in Fig. 3), even discontinued subjects were included, and their urinary m^7^Gua and 8-OHdG levels were significantly decreased at 2 weeks. By contrast, in the group of subjects who showed increased NNAL levels at some points during the smoking cessation program, the m^7^Gua and 8-OHdG levels did not coincide with the smoking cessation duration (Fig. 4).

### Correlation between the urinary levels of each biomarker

All data at 0, 2, and 8 weeks from all subjects were used for the correlation analyses. Among the exposure biomarkers, the urinary levels of nicotine, cotinine and NNAL significantly correlated with each other (Table 2).

**Table 2:** Spearman’s rank correlation coefficients of associations between urinary biomarkers of tobacco exposure, 8-OHdG, and m^7^Gua

Variable	Correlation coefficients			
	Nicotine	Cotinine	NNAL	m^7^Gua
Cotinine	0.80^**^	–	–	–
NNAL	0.61^**^	0.66^**^	–	–
m^7^Gua	0.16	0.26^**^	0.35^**^	–
8-OHdG	0.04	−0.06	−0.02	0.22^*^

^*^ indicates *p* < 0.05. ^**^ indicates *p* < 0.01

In the cases of the DNA damage biomarkers, the urinary levels of m^7^Gua were weakly related to the 8-OHdG levels. A comparison of the exposure markers with the DNA damage markers revealed that the urinary levels of cotinine and NNAL positively correlated with the level of m^7^Gua, but not with the 8-OHdG. The nicotine levels were not correlated with the m^7^Gua and 8-OHdG levels.

## Discussion

In a handful of individuals, the urinary nicotine and cotinine levels remained high after two weeks of the quit-smoking program, even though the median values were low in the results for all participants (Group 1 in Fig. 2a and b). Considering the short biological half-lives of nicotine and cotinine, a reasonable explanation is that the high levels were caused by smoking before visiting the clinic. Interestingly, among the subjects who completed the quit-smoking program, no one had such high levels of nicotine and cotinine (Group 2 in Fig. 2a and b). These high levels are probably derived from some subjects who could not continue the quit smoking program and had dropped out. Among the subjects who completed the quit-smoking program (Group 2 in Fig. 2), the urinary nicotine and cotinine levels decreased to 1.8 and 3.4% after quitting smoking for 2 weeks, and then to 1.7 and 0.2% after 8 weeks, relative to the values at the beginning of the smoking cessation program. In comparison, the NNAL levels were 31.4 and 12.3% at 2 and 8 weeks. These reduction rates were slower than those in previous reports with subjects in the United States [13, 33]. They may reflect racial differences in the metabolic rates of tobacco-specific biomarkers. In fact, Asians reportedly had slower rates of nicotine metabolism, as compared with those of Whites and Hispanics [34].

In this study, the smoking status during the quit-smoking program was confirmed by a medical interview of each subject during their visits to the clinic. As a result, 8 subjects mentioned smoking at some point during the quit-smoking program. Among them, however, the nicotine, cotinine, and NNAL levels were increased in only 1 subject. The smoking levels of the other 7 subjects did not elicit the increases in urinary biomarker levels measured during the clinical assessment. In many cases, increased levels of nicotine, cotinine, and NNAL were observed in the subjects who described themselves as non-smokers. Considering that these biomarkers are specific for tobacco smoking, some subjects might have not correctly declared their smoking status. In the quit-smoking program, the subjects visited the clinic at 2 or 4 week intervals. The smoking amounts and durations were not accurately identifiable from the clinical interview. Therefore, it is important to measure the tobacco-specific urinary biomarkers.

Urinary NNAL is a specific biomarker for tobacco exposure. It is not detected in nonsmokers, unless they are exposed to secondhand smoke [15]. Benowitz et al. [35] reported that the average NNAL levels were 183 pg/mg creatinine in the urine for 373 active smokers and 5.19 pg/mg creatinine for 228 passive smokers. In this study, the average NNAL levels of the subjects at the initial visit to the clinic were similar to those of the active smokers in the previous report. For patients who visit the hospitals or clinics every 2 or 4 weeks, NNAL is an appropriate biomarker for evaluating the smoking cessation status. In the group of the subjects with increased NNAL levels at some point, the m^7^Gua and 8-OHdG levels did not decrease in the treatment period. Smoking during the smoking cessation period may have affected these levels. By eliminating the subjects who showed increased NNAL levels at some points during the smoking cessation program, the following relationships between DNA damage markers and smoking cessation durations have become more apparent.

With regard to urinary DNA damage markers, the decreasing rates of the urinary m^7^Gua and 8-OHdG were 40.8 and 8.8% after quitting smoking for 2 weeks, and 48.1 and 2.2% after 8 weeks as compared with the beginning of the program. The decreasing rate of the m^7^Gua was slower than that reported previously (54% reduction after smoking cessation for 1 week) [36]. The reason for this difference may be due to the limited number of subjects and the low m^7^Gua levels at the beginning of cessation in the previous report. In this study, urinary 8-OHdG levels were decreased by 2 weeks of smoking cessation. However, among the subjects who completed the smoking cessation program, the reduction rate at 8 weeks was limited. Further studies are needed to confirm the sustained effects of the lower 8-OHdG levels elicited by quitting smoking. Several studies have shown reductions of urinary 8-OHdG levels upon quitting smoking [37, 38]. In one study of urinary 8-OHdG levels after quitting smoking, the 8-OHdG levels decreased by 23% at 4 and 26 weeks. There was no change in 8-OHdG levels after 4 weeks of smoking cessation [37], and thus the oxidative stress caused by smoking could be eliminated by quitting smoking for 1 month. The urinary 8-OHdG levels are widely employed as a useful biomarker for monitoring the oxidative stress status involved in cancer induction and lifestyle-related diseases. Although smoking is one of the major factors in elevated 8-OHdG levels, many other factors also increase them, such as ionizing radiation [39], environmental pollutants, lifestyle choices such as alcohol drinking, and so on [22, 40]. Better clarification of the health effects of smoking could be achieved with an assessment combination including another biomarker like m^7^Gua, which is produced by a different mechanism than that of 8-OHdG.

In a cross-sectional study, the urinary m^7^Gua levels positively correlated with cigarette smoking [41]. According to Spearman’s rank correlation coefficient (Table 2) in this study, the urinary m^7^Gua level was weakly associated with the cotinine and NNAL levels as the tobacco exposure markers. The results of our longitudinal epidemiological study were also in good agreement with the previous cross-sectional study [42].

## Conclusions

In patients participating in a smoking cessation program, the levels of urinary DNA damage markers (m^7^Gua and 8-OHdG) decreased with the duration of smoking cessation, in the same manner as the smoking exposure markers (nicotine, cotinine and NNAL). The urinary levels of cotinine and NNAL positively correlated with the m^7^Gua levels. NNAL may be an appropriate exposure marker for evaluating the smoking status of patients in a smoking cessation program.

## Data Availability

Not applicable.

## Abbreviations

NNAL: 4-(methylnitrosamino)-1-(3-pyridyl)-1-butanol
NNK: 4-(methylnitrosamino)-1-(3-pyridyl)-1-butanone
8-OHdG: 8-hydroxy-2′-deoxyguanosine
m^7^Gua: 7-methylguanine
